# The characterisation of microsatellite markers reveals tetraploidy in the Greater Water Parsnip, *Sium latifolium* (Apiaceae)

**DOI:** 10.1186/s13104-017-2528-6

**Published:** 2017-06-12

**Authors:** Naomi J. Dalton, Gavin J. Horsburgh, Deborah A. Dawson

**Affiliations:** 1 School of Biological Sciences, University of Bristol, Bristol Life Sciences Building, 24 Tyndall Avenue, Bristol, BS8 1TQ UK; 2 NERC Biomolecular Analysis Facility, Department of Animal and Plant Sciences, University of Sheffield, Western Bank, Sheffield, S10 2TN UK

**Keywords:** *Sium latifolium*, Microsatellite, Polyploid, Plant translocation, Simple sequence repeat (SSR), Simple tandem repeat (STR)

## Abstract

**Background:**

The Greater Water Parsnip, *Sium latifolium* (Apiaceae), is a marginal aquatic perennial currently endangered in England and consequently the focus of a number of conservation translocation projects. Microsatellite markers were developed for *S. latifolium* to facilitate comparison of genetic diversity and composition between natural and introduced populations.

**Results:**

We selected 65 *S. latifolium* microsatellite (MiSeq) sequences and designed primer pairs for these. Primer sets were tested in 32 individuals. We found 15 polymorphic loci that amplified consistently. For the selected 15 loci, the number of alleles per locus ranged from 8 to 17. For all loci, *S. latifolium* individuals displayed up to four alleles indicating polyploidy in this species.

**Conclusions:**

These are the first microsatellite loci developed for *S. latifolium* and each individual displayed 1–4 alleles per locus, suggesting polyploidy in this species. These markers provide a valuable resource in evaluating the population genetic composition of this endangered species and thus will be useful for guiding conservation and future translocations of the species.

**Electronic supplementary material:**

The online version of this article (doi:10.1186/s13104-017-2528-6) contains supplementary material, which is available to authorized users.

## Background

Plant translocation is a common occurrence, with an estimated 600 species of plants having been relocated as population introduction, re-introduction or augmentation [1, 2]. Whilst a tactic for large scale habitat restoration is through the planting of multiple species, translocation is also an important conservation strategy for specific plants at risk [3]. Guidance on plant translocations recommends consideration of genetic composition [4] however projects infrequently utilise genetic techniques in planning and evaluating reintroductions ([5]; although see [6, 7] as examples).

One species that has been widely translocated in the UK is *Sium latifolium* L., the Greater Water Parsnip. *S. latifolium* is a herbaceous, marginal aquatic perennial in the plant family Apiaceae, tribe Oenantheae; one of nine species within the genus, it is found across Europe and Asia [8]. With large, conspicuous, umbel inflorescences and growing to 2 m tall [9], *S. latifolium* was once a noticeable dominant in wetland areas of England, where it grows in habitats of fen, pond margins and grazing marsh ditches [10]. However, the population of *S. latifolium* has much declined over the past 40 years, due to habitat loss and change in wetland management [11]. It is now classified as ‘endangered’ on the vascular plant red list for England [12]. As a response to the marked decline in populations, conservation projects involving translocations of *S. latifolium* have occurred independently in at least seven counties of England, re-introducing the species in regions where it has been lost or declined, however the success of these translocations has been mixed.

The goal of this study was to generate a suite of microsatellite markers specifically developed for *S. latifolium* in order to evaluate and compare the genetic composition of populations, both old and new, with the view to guide practitioners in the best approaches for further translocations of this species. With many independent reintroductions it can also be used as a case study for exploring broader questions relating to genetic management of plant translocations.

## Results

Samples of *S. latifolium* were collected in May 2012 and August 2013 (Table 1), permission for sampling was obtained from the landowner of each site. Three leaflets per plant were preserved in silica gel and stored at room temperature. Prior to extraction, 10–20 mg of leaf tissue was frozen overnight at −80 °C before being homogenised at 1000 Hz for 3 min using a GenoGrinder 2000 (Spex CertiPrep, Metuchen, NJ USA). Genomic DNA was isolated employing a cetyltrimethyl ammonium bromide (CTAB) protocol [13], with the addition of 1% polyvinyl pyrrolidone (PVP) to the isolation buffer to remove polyphenols [14]. Once washed and air-dried, DNA was re-suspended in 100 µl low TE (10 mM Tris–HCl, 0.1 mM EDTA, pH 8.4) and subsequently diluted to 100 ng/µl with low TE.

**Table 1:** Details of *Sium latifolium* samples used for testing of the microsatellite primer sets and assessing the loci

Sample	Population	Location
Individual from which the microsatellite sequences were isolated		
I50	Wickhampton Marshes, Norfolk	TG 43535 05018
Samples used for PCR temperature gradient testing		
G15	Sutton Fen, Norfolk	TG 36511 22999
I08	Wickhampton Marshes, Norfolk	TG 43433 04160
Six unrelated individuals used initially to test for polymorphism		
B12	Tophill Low, East Riding of Yorkshire	TA 07754 49673
C12	Ouse Washes, Cambridgeshire	TL 49433 89016
D15	Romney Marsh, Kent	TQ 97837 31120
E33	Southlake Moor, Somerset	ST 36427 30272
F20	Cantley Marsh, Norfolk	TG 37352 03459
G10	Sutton Fen, Norfolk	TG 36881 23345
24 individuals from one population		
I01	Wickhampton Marshes, Norfolk	TG 43381 04180
I02	Wickhampton Marshes, Norfolk	TG 43318 04021
I03	Wickhampton Marshes, Norfolk	TG 43532 04032
I04	Wickhampton Marshes, Norfolk	TG 43193 03934
I05	Wickhampton Marshes, Norfolk	TG 43408 03171
I06	Wickhampton Marshes, Norfolk	TG 43471 04113
I07	Wickhampton Marshes, Norfolk	TG 43441 04132
I10	Wickhampton Marshes, Norfolk	TG 43921 04759
I11	Wickhampton Marshes, Norfolk	TG 44163 04634
I13	Wickhampton Marshes, Norfolk	TG 44177 04656
I15	Wickhampton Marshes, Norfolk	TG 43295 03952
I16	Wickhampton Marshes, Norfolk	TG 43325 04226
I17	Wickhampton Marshes, Norfolk	TG 43316 04050
I18	Wickhampton Marshes, Norfolk	TG 43291 03947
I19	Wickhampton Marshes, Norfolk	TG 43291 04157
I20	Wickhampton Marshes, Norfolk	TG 43252 03931
I22	Wickhampton Marshes, Norfolk	TG 43285 04125
I24	Wickhampton Marshes, Norfolk	TG 43295 03961
I25	Wickhampton Marshes, Norfolk	TG 44129 04558
I26	Wickhampton Marshes, Norfolk	TG 43288 04151
I27	Wickhampton Marshes, Norfolk	TG 43299 04101
I28	Wickhampton Marshes, Norfolk	TG 43250 03931
I29	Wickhampton Marshes, Norfolk	TG 43663 04256
I30	Wickhampton Marshes, Norfolk	TG 44131 04556

Identification code for each sample, site name and county of sampled population, British national grid reference for sample location

The microsatellite library was prepared from one individual sampled at Wickhampton Marshes, Norfolk, UK (52°35′N 1°35′E; sample identification code = I50). The library was enriched for microsatellites, using magnetic beads in the hybridisation [15, 16]. An Illumina paired-end library was created using 1 µg of the repeat-enriched genomic DNA. The SureSelect Library Prep Kit, ILM (Agilent Technologies Inc. Santa Clara, California) protocol was followed and 2 × 250 bp paired-end sequencing conducted using a MiSeq Benchtop Sequencer (Illumina Inc. San Diego, California).

Sequences with at least ten repeats were selected for primer design; primer sets were designed to amplify the microsatellite regions using PRIMER3 v 0.4.0 [17]. Specifications for primer selection were set at a primer length of 16–36 base pairs (optimum 20 bp), an optimal primer melting temperature of 60 °C, (min–max of 59–61 °C), a maximum of 0.5 °C between primers, presence of a 3′ GC clamp, a maximum poly-X of three and the default settings for all other parameters. Sixty-five primer sets were designed. The 5′ end of each forward primer was fluorescently-labelled with HEX or 6-FAM.

Microsatellites were amplified in 2-µl PCRs, including 1 µl (100 ng) genomic DNA (air dried), 2 µl primer mix (forward and reverse primer at 0.2 µM) and 1 µl Qiagen Multiplex PCR Master Mix including HotStar *Taq* DNA polymerase (Qiagen Inc.). Covered with a thin layer of mineral oil, products were amplified under the following profile: incubate at 95 °C for 15 min, followed by 35 cycles of 94 °C for 30 s, selected primer temperature (51, 53 or 58 °C, see Table 2) for 1 min 30 s and 72 °C for 1 min 30 s, and finally incubated at 72 °C for 10 min. The optimum annealing temperature for each primer set was initially selected by testing a temperature gradient on two samples (Table 1), this varied the annealing temperature for each well across 12 rows from 50 to 70 °C. PCR products were diluted with double-deionized H_2_O (1:160). They were visualised on an ABI 3730 48-well capillary DNA Analyser (Applied Biosystems Inc. California, USA) and sized with a ROX-labelled size standard. Allele sizes were scored using GENEMAPPER v3.7 software (Applied Biosystems Inc. California, USA).

**Table 2:** Details for the 15 selected, validated *Sium latifolium* microsatellite loci

Locus	Sequence identifier and accession no.	Primer sequences (5′–3′)	Repeat motif	T (°C)
Sla01	GWP00014, LN849725	F: [6FAM]AGACTTGTATGTCCTGCATTATGTTC R: CAGCTGGTGAAGCCAATTTAG	(GT)13	58
Sla02	GWP00025, LN849726	F: [HEX]TTGCCTCAAGTGCAGAACAG R: CAACCACTTACATATGTTCACAATACC	(CT)15	58
Sla03	GWP00030, LN849727	F: [6FAM]ACCAATGACAAGTGGGTTCC R: CCCAAGATTTCCTTGAAGTACAG	(CA)28	53
Sla04	GWP00089, LN849728	F: [HEX]GATTCCCGATCTCCAATTCC R: CGCGACATCGAAGAGTTTG	(CA)13	53
Sla05	GWP00130, LN849729	F: [6FAM]AGAAGCACGCTATTGCACTG R: CATTTGTCAGTTGTCACATACCC	(GT)10	58
Sla06	GWP00133, LN849730	F: [6FAM]TTGCAAGGAAACTGAGACCAC R: TGGACATTGTACCAGCTACCC	(CT)14	51
Sla07	GWP00178, LN849731	F: [6FAM]GGACATCTAAGCATAAAGTGCAATAAC R: TTGTTTCTAGCAGAGGTAGCTTGAC	(CA)18	58
Sla08	GWP00226, LN849732	F: [HEX]CAGATGGATAGTTGAAACCAAGTG R: TTAAGTTAGACAAGCGGCCTTC	(CA)12	51
Sla09	GWP00268, LN849733	F: [HEX]CAGCAAGAATTGCCAATCG R: AATGGTGAAGGGAAATGCTG	(GT)12	58
Sla10	GWP00318,LN849734	F: [HEX]TTACTTGCCCACGCTTCTG R: TCTTCTAAAGCAGGGGAGTACG	(CT)15	51
Sla11	GWP00319, LN849735	F: [6FAM]TGATACGGTGGATGATGAGC R: TGCATTATATGCGTCAACTGG	(GT)12 (GA)8	58
Sla12	GWP00373, LN849736	F: [6FAM]GCCACAGTAGATCCATTACTCAAC R: TTTGACACAGATTGGAATCCTC	(GT)16	51
Sla13	GWP00423, LN849737	F: [HEX]CCTTAACTAAAGACTAAAGACTGTGGAAC R: ACTTGGTCGGTTATGTTGTGG	(GA)13	58
Sla14	GWP03443, LN849738	F: [6FAM]CTGGCAAACACACGCAAC R: TTTCTTTGTTTGGGTTTGATCTC	(GA)13	58
Sla15	GWP03601, LN849739	F: [6FAM]TTGTAAACGCCCTTACCATTG R: AATAAACCATGAACAGATGAAGATTG	(GT)15	51

Microsatellite loci, sequence identifier and EMBL/EBI accession number, sequence of primers, repeat motifs, optimum primer annealing temperatures (T °C)

All primer sets were initially tested in six unrelated individuals (Table 1), each from a different geographic population in the UK. Markers failing to amplify or appearing monomorphic at this stage were discarded. The remaining primer sets were then tested in a further 24 individuals from the same population as the individual sequenced to isolate the microsatellites (Wickhampton Marshes, England; I50; Table 1) to fully evaluate their characteristics and usefulness. Overall, of the 65 primer pairs tested, 15 (23%) loci were polymorphic and easily scoreable (Table 2). The remainder were monomorphic (18%), not useable due to stutter and scoring difficulty (31%) or had poor/no amplification (28%).

To estimate genotyping error, extraction and scoring for a proportion of individuals was repeated to compare the data. The mean scoring error was found to be 0.02% (calculated as per [18]). All of the 15 markers tested displayed more than 2 alleles in multiple individuals and all individuals tested displayed more than 2 alleles in several markers, suggesting *S. latifolium* is polyploid (for data, see Additional file 1). A maximum of 4 alleles were observed per individual indicating tetraploidy in this species (see Additional file 2). Characteristics of each microsatellite locus were calculated for *S. latifolium* samples using the R package polysat [19, 20]. The number of alleles per locus ranged from 8 to 17 and the mean average number of alleles was 12 (Table 3). Observed heterozygosity per locus ranged from 0.88 to 1.00, with a mean average of 0.99 (Table 3). Due to polyploidy and unknown inheritance patterns, deviation from Hardy–Weinberg equilibrium could not be calculated nor could the frequency of null alleles be estimated [21].

**Table 3:** Characterisation of 15 dinucleotide microsatellite loci for the Greater Water Parsnip *Sium latifolium*, all tested on 24 individuals sampled at Wickhampton Marshes, reveals tetraploidy in this species

Locus	Fluoro dye	Exp. I50 (bp),	Obs. I50 (bp).	N	K	Observed allele size range (bp)	Number of individuals with 1–2 alleles	Number of individuals with 3–4 alleles	Ho
Sla01	[6FAM]	192	**191**, 193, 195	23	12	189–213	0	23	1.000
Sla02	[HEX]	154	132, 150, **154**, 164	24	17	132–180	4	20	1.000
Sla03	[6FAM]	241	230, 232, **240**	23	16	202–242	10	13	1.000
Sla04	[HEX]	196	180, 188, 194*	24	9	180–204	9	15	1.000
Sla05	[6FAM]	248	244, **248**, 250	23	8	242–254	8	15	0.958
Sla06	[6FAM]	154	130, 138, 150, **154**	23	9	130–158	5	18	0.958
Sla07	[6FAM]	228	203, 207, 224*	24	12	203–224	2	22	1.000
Sla08	[HEX]	115	104, 110, 112*	24	15	94–136	3	21	1.000
Sla09	[HEX]	180	168, 182*	24	10	166–186	21	3	0.875
Sla10	[HEX]	142	132, **141**	23	13	128–170	14	9	1.000
Sla11	[6FAM]	148	136, 142, **148**	22	11	128–156	8	14	1.000
Sla12	[6FAM]	107	92, **106**, 108, 112	22	13	90–116	3	19	1.000
Sla13	[HEX]	121	117, 128*	23	11	110–136	11	12	1.000
Sla14	[6FAM]	161	158, **160**, 168	22	14	134–176	4	18	1.000
Sla15	[6FAM]	106	101, **105**, 107, 119	23	12	83–119	7	19	1.000

Microsatellite loci, expected and observed allele sizes (with the sequenced allele underlined*; bp) of individual from which the microsatellite sequences were isolated (individual I50, sampled at Wickhampton Marshes, Norfolk), number of individuals successfully genotyped (n), number of alleles (k), allele size range (bp), observed heterozygosity (Ho). Exp. I50 (bp), Expected allele size of I50, Obs. I50 (bp), Observed amplified allele sizes of individual, I50, *Minor size differences (bp) were observed between the expected size of the allele (based on sequencing) and observed allele size (based on ABI genotyping). This error is caused by (1) the presence of the fluorescent dye label (6FAM and HEX) and/or (2) sequence misalignment due to the repeat region when creating the consensus sequence from the two paired-end complementary sequences

**Fig. 1:**
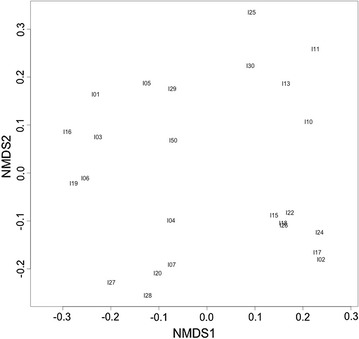
Genetic distance between 25 individuals sampled at Wickhampton Marshes, Norfolk, UK. Dissimilarity calculated on Bruvo Distance. NMDS plot stress = 0.217

Initial measures of genetic diversity were calculated for the genotyped population (Wickhampton Marshes) using the programme GenoDive [22]. In this population, the mean average number of alleles per locus was 9.13 and observed heterozygosity was 0.976. Genetic distances between individuals within the library population were calculated (Bruvo distance, R package polysat [20, 23]) and visualised by ordination (R package Vegan [24]). The microsatellite markers revealed variation in the genetic distance between individuals within a single population and identified clusters of individuals with similar genotypes (Fig. 1).

## Conclusions

We have successfully developed the first set of microsatellite markers for *S. latifolium*. The 15 loci amplified reliably and have been shown to be sufficiently variable for distinguishing between individuals (Fig. 1). These will be helpful in providing a genetic context for planning and managing further reintroductions of *S. latifolium*. Additionally, using *S. latifolium* as an example species, these microsatellite loci will also be helpful in interpreting the effects of genetic diversity and source population composition on plant reintroductions.

We also found each *S. latifolium* individual genotyped displayed 1–4 alleles. We conclude that this is evidence of tetraploidy, a trait not previously reported in this species. Polyploidy occurs occasionally through the Apiaceae family, in just over 10% of species [25]. In other species of *Sium* intraspecific variation in ploidy levels has been recorded, with local polyploid cytotypes found within a diploid species [26]. A chromosome count of 12 or 20 has been reported in *S. latifolium* [27]. As these previous cytological studies used specimens from continental Europe, the chromosomal characteristics of UK *S. latifolium* is unknown. Differences in records suggests that there may be variation within the species and all reported counts are a multiple of 4, indicating that tetraploidy is possible. Additional cytological analyses would also consider historical polyploidy or aneuploidy as causes of the multiple alleles observed. Further work on *S. latifolium* is needed to determine the nature of the ploidy (i.e. the inheritance type) and the patterns of ploidy throughout the species’ geographic range.

## Additional files

**Additional file 1.** Genotypes of tested individuals: Complete genotyping data of individuals characterised in marker selection.

**Additional file 2.** ABI electropherograms of individuals displaying tetraploidy for three markers A) Sla01, B) Sla06 and C) Sla12 (individuals were sampled at the Wickhampton Marshes, Norfolk). Sample identification codes are shown in italics.

## Abbreviations

CTAB: cetyltrimethyl ammonium bromide
DNA: deoxyribonucleic acid
EDTA: ethylenediamine tetraacetic acid
PCR: polymerase chain reaction
PVP: polyvinyl pyrrolidone
TE: Tris–EDTA
UK: United Kingdom

## References

[1] Dalrymple SE, Stewart GB, Pullin AS. Are reintroductions an effective way of mitigating against plant extinctions? CEE review 07-008 (SR32). Collaboration for environmental evidence. 2011. http://www.environmentalevidence.org/SR32.html.

[2] Godefroid S, Vanderborght T (2011). Plant reintroductions: the need for a global database. Biodivers Conserv.

[3] Maunder M (1992). Plant reintroduction: an overview. Biodivers Conserv.

[4] IUCN/SSC (2013). Guidelines for reintroductions and other conservation translocations.

[5] Weeks AR, Sgro CM, Young AG, Frankham R, Mitchell NJ, Miller KA, Byrne M, Coates DJ, Eldridge MDB, Sunnucks P, Breed MF, James EA, Hoffmann AA (2011). Assessing the benefits and risks of translocations in changing environments: a genetic perspective. Evol Appl.

[6] Gonzalez-Perez MA, Lledo MD, Lexer C, Fay M, Marrero M, Banares-Baudet A, Carque E, Sosa PA (2009). Genetic diversity and differentiation in natural and reintroduced populations of *Bencomia exstipulata* and comparisons with *B. caudata* (Rosaceae) in the Canary Islands: an analysis using microsatellites. Bot J Linn Soc.

[7] Lloyd MW, Burnett RK, Engelhardt KAM, Neel MC (2012). Does genetic diversity of restored sites differ from natural sites? A comparison of *Vallisneria americana* (Hydrocharitaceae) populations within the Chesapeake Bay. Conserv Genet.

[8] Spalik K, Downie SR (2006). The evolutionary history of *Sium* sensu lato (Apiaceae): dispersal, vicariance, and domestication as inferred from ITS rDNA phylogeny. Am J Bot.

[9] Stace C (2010). New flora of the British Isles.

[10] Preston C, Pearman D, Dines T (2002). New atlas of the British and Irish flora.

[11] Stewart ADP, Preston C (1994). Scarce plants in Britain.

[12] Stroh P, Leach S, August T, Walker K, Pearman D, Rumsey F, Harrower C, Fay M, Martin J, Pankhurst T, Preston C, Taylor I (2014). A vascular plant red list for England.

[13] Doyle JJ, Doyle JL (1987). A rapid DNA isolation procedure for small quantities of fresh leaf tissue. Phytochem Bull.

[14] Maliyakal EJ (1992). An efficient method for isolation of RNA and DNA from plants containing polyphenolics. Nucleic Acids Res..

[15] Armour JAL, Neumann R, Gobert S, Jeffreys AJ (1994). Isolation of human simple repeat loci by hybridization selection. Hum Mol Genet.

[16] Glenn TC, Schable NA (2005). Isolating microsatellite DNA loci. Method Enzymol.

[17] Rozen S, Skaletsky HJ, Misener S, Krawetz S (1999). Primer 3 on the WWW for general users and for biologist programers. Methods in molecular biology: bioinformatics methods and protocols.

[18] Hoffman JI, Amos W (2005). Microsatellite genotyping errors: detection approaches, common sources and consequences for paternal exclusion. Mol Ecol.

[19] R Development Core Team R. R (2011). A language and environment for statistical computing. R foundation for statistical. Computing.

[20] Clark LV, Jasieniuk M (2011). Polysat: an R package for polyploid microsatellite analysis. Mol Ecol Resour.

[21] Dufresne F, Stift M, Vergilino R, Mable BK (2014). Recent progress and challenges in population genetics of polyploid organisms: an overview of current state-of-the-art molecular and statistical tools. Mol Ecol.

[22] Meirmans PG, Van Tienderen PH (2004). GENOTYPE and GENODIVE: two programs for the analysis of genetic diversity of asexual organisms. Mol Ecol Notes.

[23] Bruvo R, Michiels NK, D’Souza TG, Schulenburg H (2004). A simple method for calculation of microsatellite genotype distances irrespective of ploidy level. Mol Ecol.

[24] Oksanen J, Blanchet FG, Kindt R, Legendre P, O’Hara RB, Simpson GL, Solymos P, Stevens MHH, Wagner H. Vegan: community ecology package, R package version 2.0–4. 2012. http://CRAN.R-project.org/package=vegan. Accessed 7 Apr 2016.

[25] Wood TE, Takebayashi N, Barker MS, Mayrose I, Greenspoon PB, Rieseberg LH (2009). The frequency of polyploid speciation in vascular plants. P Natl Acad Sci USA.

[26] Kumar S, Jeelani SM, Rani S, Kumari S, Gupta RC (2014). Cytological evaluation of Apiaceae Lindl from Western Himalayas. Cytol Genet.

[27] Rice A, Glick L, Abadi S, Einhorn M, Kopelman N, Salman-Minkov A, Mayzel J, Chay O, Mayrose I (2015). The Chromosome Counts Database (CCDB)—a community resource of plant chormosome numbers. New Phytol.

