# Supplementary figures and images for: The characterisation of microsatellite markers reveals tetraploidy in the Greater Water Parsnip, Sium latifolium (Apiaceae)
Source: BMC Res Notes. 2017 Jun 12;10:204. doi: 10.1186/s13104-017-2528-6 (PMC5469066; doi:10.1186/s13104-017-2528-6)

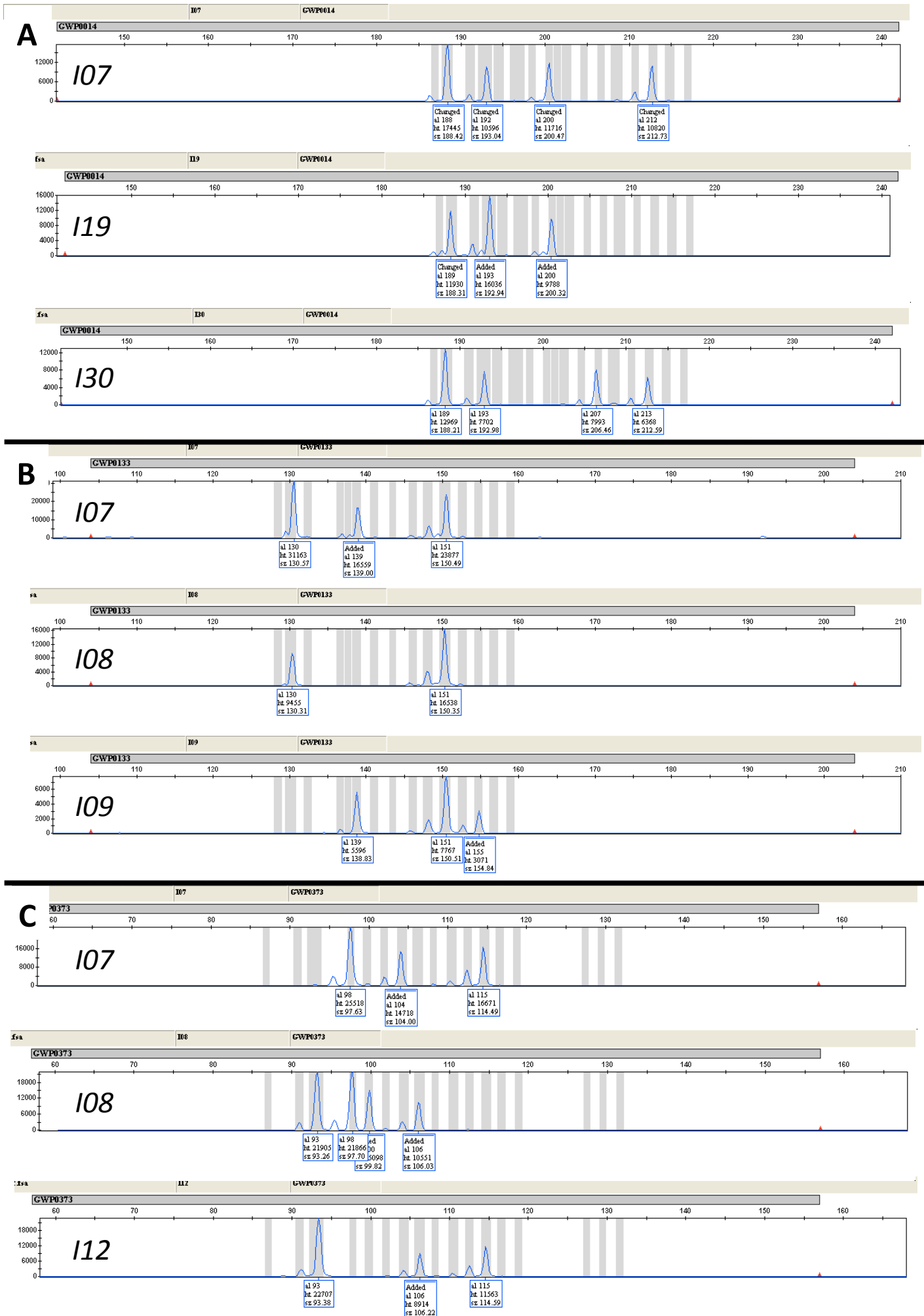

Supplement: Supplementary file 2 — Additional file 2. ABI electropherograms of individuals displaying tetraploidy for three markers A) Sla01, B) Sla06 and C) Sla12 (individuals were sampled at the Wickhampton Marshes, Norfolk). Sample identification codes are shown in italics. [file 13104_2017_2528_MOESM2_ESM.pdf]
